# Supplementary material for: Evaluating the Adoption of Evidence-Based Management Practices in Eye Hospitals
Source: Healthcare (Basel). 2025 Jan 22;13(3):222. doi: 10.3390/healthcare13030222 (PMC11817388; doi:10.3390/healthcare13030222)
Supplement: Supplementary file 1 [file healthcare-13-00222-s001.zip › healthcare-3400306-supplementary.pdf]

Study on Management Practices in eye hospitals

Participant details:

Name of the organization (not mandatory): ....

Title of the respondent: ....

Years of experience in the current hospital ....

Management practices

(*Please choose the most appropriate option in the scale that reflect your organisation*)

1. My organization believes it is important to adopt new and innovative practices.

	1	2	3	4	5	6	7	
Never	◯	◯	◯	◯	◯	◯	◯	Always

2. My organization makes decisions by looking at what other organizations are doing.

	1	2	3	4	5	6	7	
Never	◯	◯	◯	◯	◯	◯	◯	Always

3. My organization uses benchmarking to identify best practices used in other organizations.

	1	2	3	4	5	6	7	
Never	◯	◯	◯	◯	◯	◯	◯	Always

4. My organization uses consultants to help us make decisions.

	1	2	3	4	5	6	7	
Never	◯	◯	◯	◯	◯	◯	◯	Always

5. Before any (strategic / operational / routine / tactical) decision is taken we consult experienced professionals within our organisation to verify claims regarding assumed problems or effective solutions.

	1	2	3	4	5	6	7	
Never	◯	◯	◯	◯	◯	◯	◯	Always

6 .Before any (strategic / operational / routine / tactical) decision is taken, we consult the most important stakeholders (people inside or outside the organisation those may be affected by the decision) to verify claims regarding assumed problems or effective solutions.

	1	2	3	4	5	6	7	
Never	◯	◯	◯	◯	◯	◯	◯	Always

7. Before any (strategic / operational / routine / tactical) decision is taken, we systematically evaluate internal data to better understand the nature of the problem

	1	2	3	4	5	6	7	
Never	◯	◯	◯	◯	◯	◯	◯	Always

8. Managers and senior staff in my organisation have access to appropriate information systems that contain People Data (e.g. absenteeism, turnover, job satisfaction), Patients Data (Number of patients served, Patients' complaints and satisfaction) and overall Business Performance Data (e.g. productivity, financial indicators)

	1	2	3	4	5	6	7	
None of them	◯	◯	◯	◯	◯	◯	◯	All of them

9. Before any (strategic/tactical/important) decision is taken, my organisation consults the scientific literature to verify claims regarding assumed problems or effective solutions.

	1	2	3	4	5	6	7	
Never	◯	◯	◯	◯	◯	◯	◯	Always

10. Managers and senior staff in my organisation know how to use the Internet to search for scientific evidence to guide their decisions.

None of them ◯1 ◯2 ◯3 ◯4 ◯5 ◯6 ◯7 All of them

11. Managers and staff in my organisation know how to critically assess the quality of organisational / internal data.

None of them ◯1 ◯2 ◯3 ◯4 ◯5 ◯6 ◯7 All of them

12. Managers in my organisation know how to appraise the trustworthiness of the findings from scientific research.

None of them ◯1 ◯2 ◯3 ◯4 ◯5 ◯6 ◯7 All of them

13. Before we implement new policies or practices we obtain a baseline against which subsequent evaluation can be compared.

Never ◯1 ◯2 ◯3 ◯4 ◯5 ◯6 ◯7 Always

14. Our organisation systematically evaluates the effectiveness of new policies and practices before we introduce.

Never ◯1 ◯2 ◯3 ◯4 ◯5 ◯6 ◯7 Always

15. Managers in my organization tend to believe that the organization is unique and hence the outcome of scientific research is not applicable.

None of them ◯1 ◯2 ◯3 ◯4 ◯5 ◯6 ◯7 All of them

16. Managers and senior staff in my organization tend to believe that experience and knowledge gained on the job is the only important source of information when considering how to tackle a problem.

None of them ◯1 ◯2 ◯3 ◯4 ◯5 ◯6 ◯7 All of them

17. Internal politics and power struggles influence the way my organization makes decisions about policies and practices.

Never ◯1 ◯2 ◯3 ◯4 ◯5 ◯6 ◯7 Always

18. If we make mistakes in our decision-making we try to learn from them.

Never ◯1 ◯2 ◯3 ◯4 ◯5 ◯6 ◯7 Always

19. We follow a systematic improvement process to address the problems and work on improvements. eg. Lean six sigma, IHI (Institute of Health Improvement), TPM, TQM model etc.

Never ◯1 ◯2 ◯3 ◯4 ◯5 ◯6 ◯7 Always

Thank you for taking part in this survey. Your responses will be helpful for understanding of current management practices in eye hospitals.
